# Quantitative trait loci analysis of brown blotch resistance in cowpea variety KN1

**DOI:** 10.1007/s11032-018-0867-1

**Published:** 2018-08-16

**Authors:** Erik W. Ohlson, Gilles I. Thio, Mahamadou Sawadogo, Paco Sérémé, Michael P. Timko

**Affiliations:** 1 Department of Biology, University of Virginia, Charlottesville, VA 22904 USA; 2 Laboratory of Genetic and Plant Biotechnology, Institut de l’Environnement et de Recherches Agricoles (INERA), Ouagadougou, 01 BP 476 Burkina Faso; 3 Laboratory of Plant Pathology, Institut de l’Environnement et de Recherches Agricoles (INERA), Ouagadougou, 01 BP 476 Burkina Faso; 4 Laboratory of Biosciences/Genetics and Biotechnology, Université Ouaga 1 Pr Joseph Ki-Zerbo, Ouagadougou, Burkina Faso

**Keywords:** Cowpea, Brown blotch, *Colletotrichum capsici*, Disease resistance, Quantitative trait loci mapping

## Abstract

Cowpea is one of the most important crops in West Africa and is essential for the region’s food and nutrition security and economic development. Consequently, improving its agronomic performance and yield is a desirable goal. Brown blotch disease, caused by the fungal pathogen *Colletotrichum capsici*, is an important constraint of cowpea productivity, and at present, only limited genetic resources are available for breeding improved brown blotch-resistant varieties. The current study has characterized the genetic basis for brown blotch resistance conferred by the cowpea cultivar KN1 and identified a major dominant quantitative trait locus (QTL) for resistance on chromosome Vu02. A segregating F_2_ population (*n* = 200), derived from a cross between KN1 and brown blotch-susceptible Tiligre (KVx775-33-2G), was developed and scored for disease severity following controlled inoculation. A subset of the population (*n* = 94) was genotyped with 99 newly developed allele-specific polymerase chain reaction (AS-PCR) markers, and multiple interval mapping was performed. One major and three minor QTL were identified. This is the first reported mapping of QTL conferring resistance to *C. capsici* in cowpea, and it is expected that the markers identified here will be a valuable resource for developing elite cowpea cultivars with resistance to brown blotch.

**Electronic supplementary material:**

The online version of this article (10.1007/s11032-018-0867-1) contains supplementary material, which is available to authorized users.

## Introduction

Cowpea (*Vigna unguiculata* (L.) Walp.) is an important legume grown for food and fodder in the semi-arid tropics. Worldwide cowpea production is estimated at 6.5 million metric tons annually, with 80% of production occurring in West Africa (Boukar et al. 2016). Four of the world’s top five cowpea producing countries include Nigeria, Niger, Burkina Faso, and Cameroon (FAOSTAT.org). Cowpea is recognized as a key crop for food and nutrition security and economic development in sub-Saharan Africa due to its good protein content, ability to grow in and enhance marginal soils, and high drought and heat tolerance. Consequently, substantial efforts have been made to improve cowpea productivity through traditional and molecular breeding.

Cowpea production is constrained by various biotic and abiotic stresses including pests and diseases, drought, and soil fertility. An increasing pool of genomic data (Timko et al. 2008; Muñoz-Amatriaín et al. 2016; *Vigna unguiculata* v1.0, NSF, UCR, USAID, DOE-JGI, http://phytozome.jgi.doe.gov/) has facilitated the identification of genetic markers and quantitative trait loci (QTL) associated with resistance to several important cowpea pests and diseases. These pests and diseases include *Striga gesnerioides* (Ouédraogo et al. 2001, 2002b; Li and Timko 2009; Ouedraogo et al. 2012), aphids (Huynh et al. 2015; Kusi et al. 2018), thrips (Omo-Ikerodah et al. 2008; Muchero et al. 2010), root-knot nematodes (Ouédraogo et al. 2002a; Huynh et al. 2016), *Fusarium* wilt (Ouédraogo et al. 2002a; Pottorff et al. 2012), *Macrophomina phaseolina* (Muchero et al. 2011), *Cercospora* leaf spot (Duangsong et al. 2016), bacterial blight (Shi et al. 2016), and various mosaic viruses (Ouédraogo et al. 2002a). Despite significant progress developing molecular breeding tools for cowpea breeders, economically important diseases still lack genetic markers including brown blotch disease, caused by the fungal pathogen *Colletotrichum capsici* [Syd.] Butler and Bisby.

*Colletotrichum* species include some of the most devastating pathogens worldwide, causing anthracnose and anthracnose-like diseases in many important crop species. *C. capsici* has a wide host range (Pring et al. 1995; Damm et al. 2009). Isolates collected from cowpea were also able to infect other legume species including common bean, chickpea, and mung bean (Pring et al. 1995). While brown blotch has reportedly been instigated by both *C. capsici* and *Colletotrichum truncatum*, evidence suggests that in Nigeria, the majority of cases are caused by *C. capsici* (Emechebe and Florini 1997). Furthermore, recent studies of *Colletotrichum* diversity based on DNA sequence comparisons at multiple loci (e.g., ITS, ACT, Tub2, CHS-1, GAPDH, and HIS3) suggest that *C. capsici* and *C. truncatum* are highly similar and may be considered synonymous (Damm et al. 2009). The infection process by *C. capsici* occurs quickly. Conidia germinate within 16 h of inoculation, which is followed by rapid enzymatic destruction of cellular barriers. Hyphae initially propagate between epidermal and cortical cells and acervuli form within a week under favorable conditions. In later stages, after prolonged exposure to destructive enzymes, lignified cells may be directly penetrated by hyphae (Pring et al. 1995).

Brown blotch is especially prominent in the West and Central African rainforest zones, the southern Guinea savanna, and the southern part of the northern Guinea savanna (Emechebe and Florini 1997). Yield losses due to brown blotch vary by region but are severe under favorable conditions. Yield loss due to brown blotch is estimated at 46–75% in the northern Guinea savanna of Nigeria (Emechebe 1981; Alabi 1994). The disease affects all aboveground plant parts and is especially harmful to young plants (Alabi 1994). Brown blotch may also cause flower abortion and under severe infection results in vascular tissue collapse and plant death. The pathogen may be seed-borne, disrupting germination or causing damping off of seedlings. Even if seed is obtained from infected plants, it is often unmarketable due to discoloration and an increased potential for seed-borne transmission.

Based on their differential ability in eliciting a disease response, eight possible races of *C. capsici* were identified among 120 isolates collected from cowpea varieties in Nigeria (Emechebe 1986). Similarly, four genetic variants and three pathogenic groups were identified among *C. capsici* isolates collected from different agro-ecological zones in Burkina Faso based on ITS sequencing and the differential reactions of three cowpea varieties (Thio et al. 2016, 2017). The diversity reported among *C. capsici* isolates suggests that it may be necessary to employ multiple control methods against the disease. Cultural practices including growing seed from uninfected areas or intercropping limit the spreading and severity of disease (Adebitan et al. 1996). While some fungicides, such as benomyl, effectively control brown blotch disease, frequent applications are costly to growers, burden the environment, and may lead to resistant isolates. Biocontrols, such as *Trichoderma viride*, or botanicals like *Jatropha curcas* extracts, have reportedly led to significant overall reductions in disease severity (DS) (Bankole and Adebanjo 1996; Onuh et al. 2008). Additionally, soil quality improvement with supplemental phosphorus reduced DS (Owolade et al. 2006). However, breeding improved cowpea varieties with durable genetic resistance to brown blotch remains the most desirable control method.

A study of 74 cowpea varieties in the humid tropics of Nigeria reported 64–100% were susceptible to brown blotch disease (Ajibade and Amusa 2001). Similarly, evaluation of 41 cowpea varieties against highly aggressive *C. capsici* isolates collected from three agro-ecological zones of Burkina Faso suggested 76% were susceptible (Thio et al. 2017). Only KN1 (also known as Vita 7) was found to be resistant to all three isolates. Consequently, it is necessary to pursue breeding efforts to identify new sources of brown blotch resistance for use in developing resistant cowpea varieties. While resistance to brown blotch disease in cowpea has been reported (Abadassi et al. 1987; Adebitan et al. 1992; Thio et al. 2017), the overall genetic analysis of inheritance has been limited. Abadassi et al. (1987) found that brown blotch resistance was controlled by a single partially dominant gene based on F_2_ and backcross segregation. However, recessive resistance to brown blotch disease has been identified in other cowpea varieties (G. Thio, *personal communication*).

The high level of broad spectrum resistance observed in KN1 makes it an ideal candidate for breeding brown blotch-resistant cowpea varieties. However, in order to effectively select for resistance, genetic markers linked to the resistance gene are desirable to employ marker-assisted selection (MAS). An F_2_ mapping study was undertaken in order to determine genomic regions associated with resistance. Here, we report the identification of a major dominant resistance QTL and provide several PCR-based markers of use to breeders.

## Materials and methods

### Population development and disease evaluation

An F_2_ mapping population (*n* = 200) was generated from a cross between brown blotch-susceptible Tiligre (KVx775-33-2G) and the multi-race-resistant variety, KN1 (Vita 7). KN1 is a cowpea variety grown widely in Burkina Faso, and Tiligre is a new, high yielding variety developed at the Institut de l’Environnement et de Recherches Agricoles (INERA) with desirable agronomic traits including *Striga gesnerioides* resistance.

The F_2_ population was grown in D16R deepots (Stuewe and Sons, Inc.) for 14 days in a controlled greenhouse environment on a 12-h photoperiod. Temperature was maintained at approximately 24 °C. After 14 days, the population was inoculated with a suspension of *C. capsici* as described below.

A *C. capsici* single-spore isolate collected from Saria (SA) in Burkina Faso was used for disease evaluation. The isolate was cultured on potato dextrose agar (PDA) and incubated at 24–28 °C for 1 week before inoculum preparation. The inoculum was prepared by submerging 10–15 mm diameter fungal disks in sterile water and vortexing to dislodge the conidia. The inoculum was filtered through sterile cheesecloth, and the conidia concentration was adjusted to approximately 10^6^ spores/ml as quantified by hemocytometer. The inoculum was applied evenly to the entire F_2_ population and parental and F_1_ controls using a handheld pump sprayer. A clear plastic screening enclosure was used to encompass the entire screening population and high relative humidity was maintained (> 60%) using a small humidifier. The plants were evaluated 28 days after inoculation and scored on a scale of 0–4 where 0 = asymptomatic, 1 = isolated spots on stem, 2 = coalesced spots, 3 = coalesced spots and visible acervuli, and 4 = withered stem or deceased plant.

### DNA collection and genotyping

Leaf tissue was collected from each plant and dried at 35 °C for 24–48 h. DNA was extracted using a modified CTAB protocol (Doyle and Doyle 1987). Both parents were previously genotyped with the Cowpea iSelect Consortium SNP Array, and 99 SNPs were converted to allele-specific PCR (AS-PCR) markers. The AS-PCR markers were developed as described by Gaudet et al. (2007) and are distinguishable from their corresponding designation on the SNP array by the prefix “A” (Table S1). A population of 94, semi-randomly selected F_2_ individuals were genotyped with the 99 AS-PCR markers. Some of the 200 F_2_ individuals were excluded due to insufficient DNA quantity or suspected derivation from incompletely inbred parental lines. Primer sequences and PCR conditions are provided in supplemental Table S1. PCR was performed in 10-μl reaction volumes consisting of 1× *Taq* buffer, 200 μM dNTPs, 25 ng genomic DNA, 0.5 μM of each primer, variable MgCl_2_ (Table S1), and 0.5 units *Taq* polymerase. PCR consisted of 2 min initial denaturation (95 °C), followed by 35 cycles of 30 s denaturation, 30 s annealing (45–60 °C; Table S1), and 30 s extension (72 °C), with a 2-min final extension (72 °C). All genotyping was visualized by 6% polyacrylamide gel electrophoresis (PAGE) stained with ethidium bromide, using a C.B.S. Scientific Mega-Gel System.

### Genetic and QTL mapping

Genetic mapping was performed in QTL IciMapping 4.1 using the default parameters (Meng et al. 2015). Markers were anchored, and linkage groups were oriented based on the cowpea reference genome (*Vigna unguiculata* v1.0, NSF, UCR, USAID, DOE-JGI, http://phytozome.jgi.doe.gov/). QTL mapping was performed in Windows QTL Cartographer 2.5 (Wang et al. 2012) using multiple interval mapping (MIM) (Li et al. 2006). The initial MIM model was developed using forward stepwise selection of markers and Bayesian Information Criterion (BIC) *g* (*n*) = ln (*n*), where *g* (*n*) is a function of sample *n*. The model was refined over multiple iterations using a walk speed of 1 centimorgan (cM) and window size of 10 until all statistically significant QTL were identified.

Visual representations of chromosomes and logarithm of the odds (LOD) scores were created in MapChart 2.3 (Voorrips 2002).

### Statistical analysis

Statistical analysis was performed using IBM SPSS Statistics (IBM Corp. 2016. IBM SPSS Statistics for Windows, Version 24.0. Armonk, NY: IBM Corp.).

## Results

### Parental, F_1_, and F_2_ disease responses

**Fig. 1:**
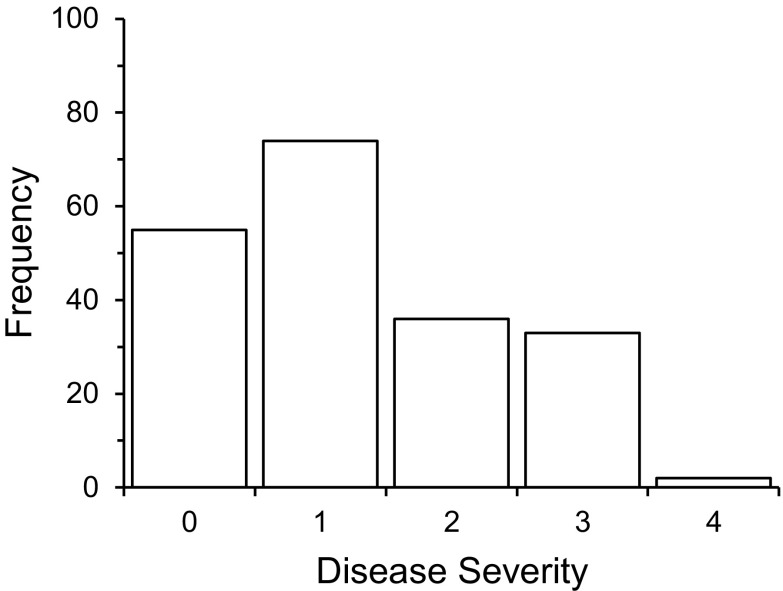
Distribution of F_2_ disease severity. Individuals were scored based on stem disease severity on a scale of 0–4, where 0 corresponds to no observed disease symptoms, 1 = small spots, 2 = coalesced spots, 3 = visible acervuli, and 4 = withered stem/deceased plant

Disease severity in the susceptible parent ranged from 1 to 4 and averaged 3.1 ± 1.1. No disease was observed on any of the KN1 or F_1_ controls. In the F_2_ generation, DS ranged from 0 to 4, averaging 1.3 ± 1.1. The F_2_ population was positively skewed towards the resistant parent (skewness = 0.5 ± 0.2), and nearly double the number of individuals were scored 0–1 compared to those scored 2–4 (Fig. 1).

### Genetic mapping and QTL analysis

**Fig. 2:**
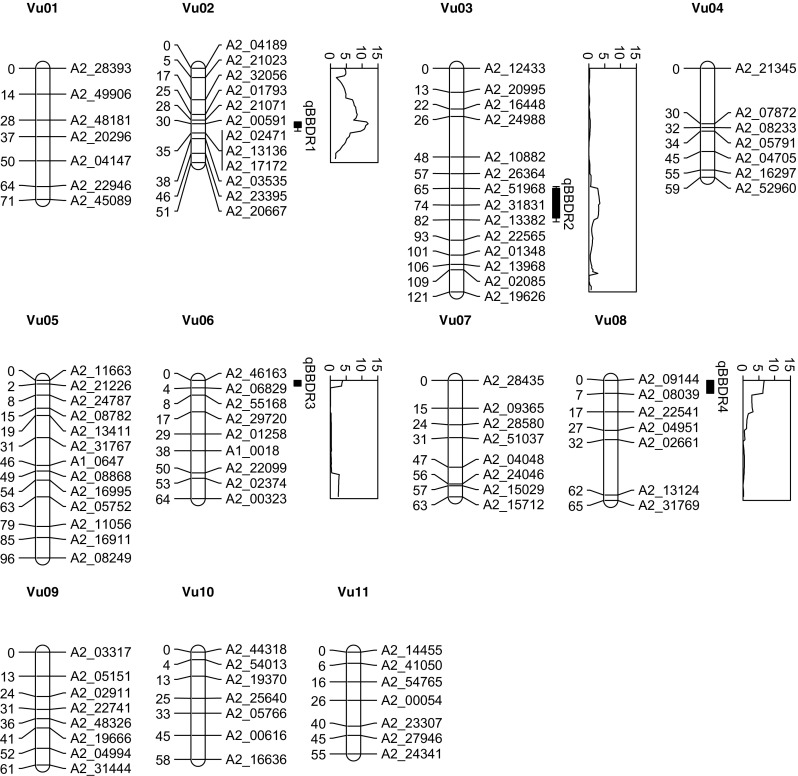
Mapping of brown blotch disease resistance in an F_2_ population derived from cowpea cultivars Tiligre and KN1. The position of each marker is provided in centimorgans (cM). One and two LOD confidence intervals are indicated for each QTL

The genetic map consisted of 99 markers and 11 linkage groups, corresponding to the 11 cowpea chromosomes (Vu01–Vu11). The map size was 763.4 cM and averaged 7.7 cM between markers, indicating good overall coverage. However, gaps of 29.5 cM were identified on Vu04 and Vu08 (Fig. 2).

The MIM model indicated the presence of four QTL located on chromosomes Vu02, Vu03, Vu06, and Vu08, which we designated qBBDR1, qBBDR2, qBBDR3, and qBBDR4, respectively. The QTL with largest effect, qBBDR1, and two minor QTL, qBBDR3 and qBBDR4, had positive additive effects (i.e., higher DS associated with the susceptible parent), while the minor QTL, qBBDR2, was slightly negative (Table 2).

qBBDR1 mapped to the proximal portion of Vu02 and had an estimated position of 30 cM. A two-LOD confidence interval situated qBBDR1 between markers A2_21071 and A2_02471. The QTL was highly significant reaching a maximum LOD of 11.9, and the additive effect was substantially higher than any of the three minor QTL. Consistent with the low DS in the F_1_ generation, the resistance was highly dominant and the effect was estimated to be − 1.5 (Fig. 2; Table 2). Examination of F_2_ segregation at the locus most tightly linked to qBBDR1 (A2_00591) indicated among individuals scoring 0–1 that only a single individual derived both alleles from the susceptible parent, while among individuals scoring 2–4 just six individuals derived one or both alleles from the resistant parent.

qBBDR2 had the smallest additive effect of any QTL identified in this study and was the only QTL for which the susceptible parent was associated with improved disease resistance. Located at 73 cM near the distal portion of Vu03, qBBDR2 was flanked by markers A2_26364 and A2_22565 (Fig. 2). While the additive effect was nearly zero and the peak LOD was just 3.5, the dominance effect was 0.93 (Table 2).

The final two QTL, qBBDR3 and qBBDR4, mapped to the tops of Vu06 and Vu08, respectively (Fig. 2). The additive effects were both higher than was calculated for qBBDR2, but approximately 10-fold smaller than was determined for qBBDR1. The dominant effect of qBBDR3 was similar to that of qBBDR2, while for qBBDR4, the effect was 1.25. The peak LOD for qBBDR3 and qBBDR4 were 3.9 and 6.7, respectively (Table 2).

## Discussion

Phenotyping segregating populations for fungal disease resistance can be challenging due to potentially large environmental effects and the presence of host quantitative disease resistance genes. Despite these challenges, there was a clear delineation of mean DS observed between Tiligre and KN1 (Table 1). While in Tiligre, the average DS exceeded 3; in KN1 and the F_1_ generation, no disease symptoms were observed. Although there was a clear distinction in mean DS between the two parents, the F_2_ generation was more quantitative, ranging from 0 to 4 and averaging slightly below the mid-parent value. Thus, although the F_1_ DS suggests the resistance conferred by KN1 is highly dominant, it also appeared that several QTL with smaller effects contributed to the quantitative F_2_ distribution. However, nearly double the number of individuals scored 0–1 compared to 2–4, suggesting the presence of at least one major dominant resistance gene, which was confirmed by QTL analysis.

**Table 1:** Disease severity of parental, F_1_, and F_2_ cowpea. Cowpea were scored on a scale of 0–4 where 0 = asymptomatic, 1 = small spots on stem, 2 = coalesced spots, 3 = visible acervuli, 4 = withered stem/deceased plant

Cultivar/population	Number of plants	Avg DS ± Std dev ^a^	Range
Tiligre	10	3.1 ± 1.1	1–4
KN1	10	0 ± 0	0
F_1_	4	0 ± 0	0
F_2_	200	1.3 ± 1.1	0–4

^a^The average disease severity ± the standard deviation

The genetic map size of 763.4 cM was consistent with previous maps of cowpea, although slightly smaller than those reported recently (Muñoz-Amatriaín et al. 2016). Overall genome coverage was generally consistent despite the relatively few markers used for mapping. For most breeding purposes, only major effect QTL are desirable, all of which would have been identified based on the high linkage disequilibrium in F_2_ populations, good average marker density, and few overall gaps in this map (Fig. 2). The 99 genome-wide SNP markers converted to simple PCR markers used in this study may also be useful for cowpea breeders who have limited access to more expensive SNP genotyping platforms (Table S1).

A MIM approach was taken in order to identify QTL associated with brown blotch resistance. MIM has improved statistical power for detecting multiple QTL compared to interval mapping approaches and has been adapted for ordinal data (Zeng et al. 2000; Li et al. 2006). Four statistically significant QTL were identified, among which the QTL qBBDR1 on chromosome Vu02 was most highly significant and had the largest additive effect. (Table 2). Tightly linked SNP markers A2_21071, A2_00591, and A2_02471, corresponding to a 7-cM interval, will be a useful tool for breeders interested in applying MAS for selecting resistance to brown blotch disease (Fig. 2). Additional SNPs previously identified in this genomic interval (Muñoz-Amatriaín et al. 2016) could also be converted to PCR markers if further markers were needed for fine mapping or introgression into other susceptible genetic backgrounds. Although the genetic distance between flanking markers was approximately 7 cM, a 1-LOD confidence interval delineated qBBDR1 to a 3-cM interval from 29 to 32 cM on Vu02. Interestingly, bulked segregant analysis of the most brown blotch-resistant and susceptible F_2_ cowpea derived from crosses KVx61-1 × Moussa Local and KVx396-4-5-2D × Donsin Local indicated that markers within the qBBDR1 interval were also highly associated with resistance conferred by Moussa Local and Donsin Local (EWO and MPT unpublished data).

**Table 2:** Four quantitative trait loci (QTL) detected in an F_2_ population derived from brown blotch-susceptible Tiligre and resistant KN1 cowpea cultivars

QTL	Chromosome	Position (cM)^a^	Additive effect^b^	Dominant effect^c^	Maximum LOD^d^
qBBDR1	Vu02	30	1.78	− 1.48	11.9
qBBDR2	Vu03	73	− 0.03	0.93	3.5
qBBDR3	Vu06	0	0.18	0.82	3.9
qBBDR4	Vu08	0	0.16	1.25	6.7

^a^Position of the QTL in centimorgans (cM)

^b^Positive values indicate increased disease severity (DS) associated with Tiligre alleles and negative values indicate increased DS associated with KN1 alleles

^c^Positive values indicate Tiligre alleles are dominant and negative values indicate KN1 alleles are dominant

^d^The maximum logarithm of the odds within the QTL

Between the qBBDR1 flanking markers, there are approximately 300 annotated genes distributed over a physical distance of slightly under 3 Mb (*Vigna unguiculata* v1.0, NSF, UCR, USAID, DOE-JGI, http://phytozome.jgi.doe.gov/). Among these genes, at least 20 are disease resistance or putative disease resistance homologs of *Arabidopsis* genes including EDR2, which is known to mediate pathogenic fungi resistance (Tang et al. 2005). Several *Colletotrichum lindemuthianum* QTL have also been reported on chromosome 2 of *Phaseolus vulgaris* L., a close relative of cowpea, suggesting this chromosome could play an important role in conferring resistance to *Colletotrichum* species (Geffroy et al. 2000; Campa et al. 2014; Oblessuc et al. 2014; Zuiderveen et al. 2016). However, fine mapping and functional analyses are needed to further characterize qBBDR1.

The remaining three brown blotch resistance QTL reported in this study appear to have less utility for breeders due to their small additive effects and are likely more environmentally specific compared to qBBDR1. However, they may partially explain the quantitative distribution of the F_2_ DS. While the F_2_ DS was skewed towards the resistant parent, there remained a large number of intermediary disease values that may be accounted for by small effect or environmentally specific resistance QTL. These results emphasize the importance of incorporating multiple disease resistance genes to achieve complete and durable resistance to brown blotch disease.

Despite its importance in West Africa, few sources of brown blotch resistance have previously been described. Here we report the identification of the first major, dominant brown blotch resistance QTL in cowpea and provide several PCR markers suitable for MAS. KN1 is a promising source of germplasm for brown blotch resistance breeding, providing nearly complete resistance to at least three highly virulent *C. capsici* isolates (Thio et al. 2017). However, it is unknown whether qBBDR1 confers resistance to all three isolates or if KN1 contains other major brown blotch resistance genes. The identification of a strong, dominant resistance QTL and several linked markers will allow breeders to quickly incorporate qBBDR1 into new elite cowpea cultivars. However, additional sources of brown blotch resistance are needed to develop elite cultivars with durable resistance. Furthermore, confirmation of qBBDR1 in different brown blotch-susceptible genetic backgrounds and across multiple environments is desirable to ensure its stability.

## Electronic supplementary material

ESM 1 (XLSX 20 kb)
